# Developing a nationwide registry of UK veterans seeking help from sector charities—a machine learning approach to stratification

**DOI:** 10.1093/eurpub/ckae141

**Published:** 2024-09-09

**Authors:** Giuseppe Serra, Marco Tomietto, Andrew McGill, Matthew Kiernan

**Affiliations:** Department of Nursery, Midwifery and Health, Northumbria University, Newcastle upon Tyne, United Kingdom; Department of Medicine (DMED), University of Udine, Udine, Italy; Department of Nursery, Midwifery and Health, Northumbria University, Newcastle upon Tyne, United Kingdom; Department of Nursery, Midwifery and Health, Northumbria University, Newcastle upon Tyne, United Kingdom; Department of Nursery, Midwifery and Health, Northumbria University, Newcastle upon Tyne, United Kingdom

## Abstract

The assistance to veterans in the UK is provided by the National Health Service and over 1800 military charities. These charities count services using different definitions and reporting systems, so to date a national registry of service usage does not exist. The aim of the Map Of Need Aggregation ResearCH study is to build a standardized registry of service usage data for the military charity sector. Data are completely anonymized by adopting a Secure Hashing Algorithm. A unique anonymous identifier is generated allowing both privacy protection and avoiding double counts. Data are standardized and linked with an automated process to create an aggregated dataset. The dataset describes the population, using both *a priori* and machine learning approaches. To date a total of 42 509 veterans with 128 423 needs are included. The mean age was 60.1 years, and 90% were male. 65% were receiving other benefits, 5% were homeless and 1% were in prison. 65% of the needs recorded concerned social wellbeing. 40% of veterans received assistance in at least two different years. The k-means clustering approach returned 4 subgroups of use that were identical to those created using *a priori* knowledge. The dataset is the most comprehensive source of veteran charity usage data in the UK to date. Service usage is generally homogenous among subgroups, but some differences were highlighted indicating that younger, non-officer veterans may be more at risk of presenting with more complex needs. These first useful insights can help allocate resources to build an effective preventive strategy for more complex cases.

Key points

- The MONARCH dataset is the first comprehensive nationwide registry in the UK of military charity data
- Previous issues with this data such as double counting were resolved using an innovative method of anonymization
- The understanding of usage patterns can lead to the design of targeted preventive strategies

## Introduction

### Veterans as a vulnerable population

There is little information on the prevalence of complex needs in veterans. Those that do experience multifactorial difficulties when they leave the military should be considered as vulnerable, as they generally have interconnected mental, physical, and social health problems [1]. Besides the risks directly connected to service (physical [2], chemical [3], biological [4]) that may cause health consequences over a long period of time, some veterans face numerous challenges upon their return to civilian life. They can suffer from mental health conditions such as PTSD, depression, and anxiety [5, 6], and face many social challenges after discharge, for instance economic hardship, unemployment, homelessness, and food insecurity [7].

These physical, mental, and social health issues often present simultaneously in a single veteran, interacting and exacerbating one another [8]. Some examples of these interactions are unemployment and financial hardship that increase the risk of many mental health conditions [9]; mental health conditions such as depression can increase the risk of hypertension, dyslipidaemia, and diabetes [10, 11]. However, survival studies have demonstrated a longer life expectancy for veterans [12] compared to general population. This effect is well-known as soldiers are pre-selected amongst physically performing individuals and this leads to an initial advantage known as healthy soldier effect (HSE). Interestingly, there is increasing evidence that this effect is eroding in recent years, bringing veteran mortality closer to that of general population [13]. Moreover, while younger veterans have a higher mortality, older veterans keep showing a clear HSE, and this may be due to a higher prevalence in younger generations of obesity [14] and unhealthy behaviour such as smoking, drinking, and drug use [15].

### Assistance for veterans in the UK

Despite the complexity of veterans’ needs, it is hard to find comprehensive data in this sector in the United Kingdom (UK) [16]. For health issues, veterans are cared for by the National Health Service (NHS), but there is no reliable national marker to identify their military veteran status [17].

In addition, Military charities in the UK provide a parallel form of assistance, ranging from income support to private doctor appointments or direct mental health support. It is, therefore, clear that studying the health of veterans while relying solely on NHS data provides an incomplete picture for this population and its complexity.

Usage data from the UK Military charities sector is fragmented and recorded in a broad range of ways that make it difficult to use. An additional challenge is that as of 2020, there were 1843 military charities in the UK, ranging from major national charities to small local ones [18]. Many of these charities provide different services as well as supporting veterans vicariously through other charities, creating a risk of over-counting service delivery.

An initial study that investigated the potential to use charity data to explore the wellbeing of veterans was undertaken by the Northern Hub for Veterans and Family Research at Northumbria University as part of the Map of Need project [7]. While it provided some insights about veterans’ health in the UK, it covered one major charity and relied on a static dataset [19]. The Map of Need project also mapped veterans' charity engagement across the UK, but data was limited to users interacting with an app-based signposting platform.

To provide a more comprehensive and self-updating source of data, the Map Of Need Aggregation ResearCH (MONARCH) project was established. The aim of this study was to build an aggregated self-updating dataset, collecting, aggregating, and making accessible charity usage data of veterans nationwide.

The aim of this article is the exploration and description of the MONARCH project data, providing the first comprehensive picture of charity service usage by veterans in the UK.

A secondary objective is to stratify the veterans according to their characteristics, in order to identify possible differences in usage patterns among subgroups.

## Methods

### Study design

Cross-sectional study.

#### Study population

The MONARCH project data consists of the veterans who have accessed military charity services between 1 January 2018 and 25 June 2023 (date of last extraction). The dataset included five of the major national charities in the UK, which covers support for all branches of the British Armed Forces. The largest of those organizations undertakes case management for over 128 smaller charities or military charity funding bodies.

#### Anonymizing data and assigning a unique identifier

The first step of linking the datasets was to assign a unique identifier for the people assisted throughout all organizations, and, for this purpose, a Secure Hash Algorithm (SHA) was used as implemented by Tomietto *et al*. [20]. This approach uses three variables to generate a 64-digit code, namely service number, date of birth, and gender. Hashing algorithms are deterministic, so they always return the same output for a given input, and this makes them vulnerable to dictionary attacks [21]. To further secure anonymization, a ‘salt’ variable (a password only known to data providers) was added as a fourth input making dictionary decrypting impossible. The identifying variables are then removed from the dataset before it is made available for analysis, leaving only the completely anonymous unique hash identifier This approach is used by the UK Office for National Statistics for anonymizing personally identifiable information [22].

### Measures

#### Definition of need and aggregation

The statistic unit of this registry was each ‘need’ captured by the charities, defined as a request, by a given person, for a given reason, in a given year, for which the veteran received assistance.

The types of needs were then classified using 3 layers of aggregation:

1. A category of need as provided by the charities
2. An aggregation based on the classification of needs used by the reports of the Dual Diagnosis National programme, specifically: family or relationship difficulties, mental health issues, social isolation, substance misuse issues, physical health, a history of offending behaviour, a learning disability, a physical disability, domestic violence, employment problems, homelessness or housing issues, poverty [23]
3. A further aggregation based on the three pillars of health (physical, social, and mental) according to the definition of the WHO [24]

#### Outcome variables

The main outcome variables are the number of needs met, the number of accesses and the repeated request for the same form of assistance. The number of accesses was computed, for each veteran, counting the number of years in which the same subject was helped. The repetition of need was assessed, for each subsequent access, checking if a need was met at least once in previous years.

#### Exposures

The characteristics of the veterans recorded by the charities were linked and harmonized:

- Age was calculated as the difference between the date of the first need recorded and the date of birth. Age was then classified in ‘working age’ (≥66 years) and ‘non-working age’ (<66 years).
- For the variables ‘gender’, ‘rank’, ‘accommodation’ ‘marital status’, ‘service’ and ‘in receipt of other benefits’, the last value to be declared was taken into consideration.
- People recorded both as a veteran and as a family member (i.e. spouse of a veteran which is also a veteran) were classified as a veteran.

### Statistical method

The statistical analysis consisted of a description of the population, of service usage and a stratification according to different usage patterns. Categorical variables were reported as absolute frequencies and percentages.

Stratification by outcome occurrence was performed in two subsequent steps: first it was done by *a priori* knowledge, dividing the population in four subgroups based on the ones provided by charities. Then, a K-means technique was used to create clusters. The results of the two approaches were then compared. In this way, it was possible to test the performance of the clusters created with the machine learning approach (K-means) against the stratification made by *a priori* assumptions. In other words, the K-means algorithm was tested to determine its effectiveness in uncovering a previously hypothesized pattern.

For the classification ‘*a priori*’, veterans assisted only in one year were further divided by the number of needs met (single vs multiple). Veterans assisted in two or more years were further divided in a subgroup always presenting with different needs (called ‘all new needs’) and a subgroup that came back at least once with the same request made in previous years (called ‘repeated need’). This led to the formation of four subgroups:

- **Single access, single need.** People that accessed a charity with a single request and did not come back. In this case, it is possible that the problem was simple, and it was solved by the access.
- **Single access, multiple needs.** People that accessed a charity with multiple requests and did not come back. In this case, it is possible that the problem was more complex than the previous scenario, but it was probably solved as the veteran did not come back.
- **Multiple access, repeated needs.** People that accessed a charity with one or more problems and came back at least in one different year with the same issue. In this case, it is possible that the effectiveness of the intervention was not optimal, as the problem kept representing over time.
- **Multiple access, all new needs.** People that accessed a charity with one or more problems and kept coming back in different years with always different requests. In this case, it is possible that the requests of services are a symptom of a deeper problem that may not be properly addressed by service providers.

For the classification based on machine learning, a K-means technique was used to create clusters of use based on: the total number of accesses, the total number of needs and the number of years in which each single need was met. K-means cluster analysis is an unsupervised machine learning technique that is useful for identifying latent patterns within a sample distribution and providing descriptions of these patterns and subgroups. To determine the ideal number of subgroups, a two-step cluster analysis was conducted, utilizing silhouette measures of cohesion, and employing the Euclidean method [25].

The Chi-Square test was adopted to assess the statistical significance of the differences among subgroups. A *P* values < .05 stated an adequate statistical significance.

The analyses were conducted using SAS Enterprise Guide version 8.2 [26] and SPSS version 28 [27].

### Ethical and data management considerations

Data management was structured according to the General Data Protection Regulation (2018) and the UK Data Protection Act (2018). Electronic data were securely stored in a protected folder accessible to the research team. Ethical approval was obtained (ref: 2055, date: 12/01/23). A data agreement was signed and legally approved between the research centre and each charity. Each dataset was then aggregated into an anonymized dataset. The aggregated registry is managed by the research centre and is accessible to each charity. Access to the aggregated data will be possible through an interactive portal in SAS Visual Analytics.

## Results

The database to date consists of 42 264 veterans and 113 521 needs. The needs concerned: 74 037 (65.2%) social health, 664 (0.6%) mental health, and 38 820 (34.2%) physical health.

Table 1 shows the characteristics of veterans who accessed charities for various needs. The majority of the veterans were male (88.6%), working age (60.9%), non-officer rank (97.7%), single/divorced/widowed (61.8%), and served in the army (64.4%). The most common type of accommodation that beneficiaries lived in was a house (49.0%), followed by flat or an apartment (27.3%) and 371 veterans (1.0%) reported living in prison. The majority of the veterans were in receipt of other benefits (64.2%) and had one or more dependants (74.0%). Most of the veterans were helped for social well-being (79.2%), roughly one third for a physical health need (35.0%), and some for a mental health need (1.2%). 60.1% did not return for further assistance, while the remaining 39.9% came back in more than one year, suggesting ongoing need. Around half of the veterans with a single access (37% of total) only reported one need, while the rest (23.1% of total) reported multiple needs. Of the veterans that were helped in more than 1 year, a small number presented with all new needs (6.8%) and a third returned for further assistance for needs that they were already helped with (33.1%). Overall, 29.4% of the users only had one need, 27.2% two needs, 15.9% three needs, 9.4% four needs, and 18.2% five or more needs. In summary, around 40% of veterans came back in two or more years, and around 70% presented with more than one need in the study period.

**Table 1.** Characteristics of veterans accessing charities

	Frequency	Percentage
**Gender**		
Female	4802	11.4
Male	37 453	88.6
Other	9	0.0
**Age**		
Non-Working Age	16 540	39.1
Working age	25 724	60.9
**Rank**		
Non-Officer	29 550	97.7
Officer	709	2.3
**Marital status**		
Married/Cohabiting/Civil	16 159	38.2
Single/Divorced/Widowed	26 105	61.8
**Service**		
Army	27 280	64.4
Other	1361	3.2
Royal Air Force	8602	20.4
Royal Marines	860	2.0
Royal Navy	4195	9.9
**Accommodation**		
Flat/apartment	10 398	27.3
Homeless/Tent/Shelter	2178	5.7
Hotel	578	1.5
House	18 673	49.0
Nursing Home	654	1.7
Other	5233	13.7
Prison	371	1.0
**In receipt of other benefits**		
No	12 553	35.8
Yes	22 529	64.2
**N. of dependants**		′
No dependants	9194	26.0
1 dependant	6552	18.5
2 dependants	9189	26.0
3+ dependants	10 449	29.5
**Type of need**		
Helped for physical health need	14 781	35.0
Helped for mental health need	495	1.2
Helped for social well-being	33 467	79.2
**Accesses per veteran**		
Did not come back (1 need)	12 409	37.0
Did not come back (multiple needs)	13 018	23.1
Came back (All new needs)	2880	6.8
Came back (Repeated needs)	13 957	33.1
**Needs per veteran**		
1	12 409	29.4
2	11 493	27.2
3	6714	15.9
4	3968	9.4
5 or more	7680	18.2

Table 2 compares the characteristics of veterans who accessed charities for various needs by usage pattern. The results show that the following characteristics were significantly associated with the usage pattern: age, rank, marital status, service, accommodation, and receipt of other benefits; the variables gender, number of dependants, type of need, and needs per veteran were not.

**Table 2.** Characteristics of veterans accessing charities by *n*. of accesses

	Multiple access, all new needs		Multiple access, repeated needs		Single access, single need.		Single access, multiple needs.		*P*
	Frequency	Percentage	Frequency	Percentage	Frequency	Percentage	Frequency	Percentage	
**Gender**									.1343
Female	310	10.8	1662	11.9	1372	11.1	1458	11.3	
Male	2570	89.2	12 294	88.1	11 033	88.9	11 556	88.6	
**Age**									< .0001
Non-working	734	25.5	6485	46.5	4887	39.4	4434	34.1	
Working age	2146	74.5	7472	53.5	7522	60.6	8584	65.1	
*Mean age*									
**Rank**									.041
Non-officer	1930	98.3	9860	97.4	8733	97.6	9042	97.9	
Officer	33	1.7	259	2.6	214	2.4	194	2.1	
**Marital status**									< .0001
Married/Cohabiting/Civil	1029	35.7	5290	37.9	4952	39.9	4888	37.6	
Single/Divorced/Widowed	1851	64.3	8667	62.1	7457	60.1	8130	62.5	
**Service**									< .0001
Army	1986	69.0	8460	60.7	8319	67.2	8443	64.9	
Other	84	2.9	453	3.3	406	3.3	418	3.2	
Royal Air Force	484	16.8	3214	23.0	2277	18.4	2627	20.2	
Royal Marines	49	1.7	243	1.7	270	2.2	298	2.3	
Royal Navy	275	9.6	1579	11.3	1116	9.0	1225	9.4	
**Accommodation**									< .0001
Flat/apartment	743	28.7	3277	26.9	2974	25.8	3404	29.0	
Homeless/Tent/Shelter	177	6.9	612	5.0	624	5.4	765	6.5	
Hotel	48	1.9	192	1.6	141	1.2	197	1.7	
House	1256	48.5	5821	47.7	5896	51.1	5700	48.5	
Nursing Home	24	0.9	294	2.4	198	1.7	138	1.2	
Other	309	11.9	1890	15.5	1593	13.8	1441	12.3	
Prison	34	1.3	108	0.9	114	1.0	115	1.0	
**In receipt of other benefits**									< .0001
No	774	31.4	3330	33.3	4258	38.9	4191	35.9	
Yes	1689	68.6	6670	66.7	6677	61.1	7493	64.1	
**N. of dependants**									.9044
No dependants	644	25.9	2599	25.7	2951	27.0	3000	25.3	
1 dependant	459	18.5	1882	18.6	1970	18.0	2241	18.9	
2 dependants	643	25.9	2671	26.4	2795	25.6	3080	26.0	
3+ dependants	738	29.7	2975	29.3	3220	29.4	3516	29.8	
**Type of need**									
Helped for physical health need	1006	34.9	5527	39.6	3924	31.6	4324	33.2	< .0001
Helped for mental health need	35	1.2	163	1.2	126	1.0	171	1.3	.1769
Helped for social well-being	2412	83.8	10 873	77.9	9657	77.8	10 525	80.9	< .0001
**Needs per veteran**									< .0001
1	0	0.0	0	0.0	12 409	100.0	0	0.0	
2	1034	35.9	2301	16.5	0	0.0	8158	62.7	
3	914	31.7	2572	18.4	0	0.0	3228	24.8	
4	532	18.5	2303	16.5	0	0.0	1133	8.7	
5 or more	400	13.9	6781	48.6	0	0.0	499	3.8	

In particular, veterans in working age tend to present multiple needs and both single and multiple accesses. Those being married tend to have single access and single or multiple needs. On the contrary, veterans declaring being not married/not co-habiting tend to have multiple accesses and present multiple needs. Moreover, veterans with multiple accesses with multiple or repeated needs have the highest frequency of living in precarious forms of housing (Table 2).

Table 3 compares the clusters made ‘*a priori*’ and the clusters computed with the k-means technique. By applying the silhouette measures of cohesion, the ideal number of clusters was found to be 4, and the clusters computed by the unsupervised approach were exactly correspondent to the ones created ‘*a priori*’. This means that each subject was grouped in the same way by the unsupervised algorithm and by the researchers, with a complete correspondence.

**Table 3.** Clusters by pattern of access

Clusters				
	1	2	3	4
Came back (All new needs)	0	0	0	2880
Came back (Repeated needs)	13 957	0	0	0
Did not come back (1 need)	0	12 409	0	0
Did not come back (multiple needs)	0	0	13 018	0

## Discussion

Our findings show that veterans accessing charities tend to be younger than the general veteran population (61% in working age vs 47%). Most notably, the gender distribution of military charity usage was similar to the general veteran population (11% female vs 13%) [28] which counters the current narrative in the UK, that women veterans face barriers to accessing services from military charities [29, 30]. This may indicate that younger veterans, irrespective of gender, are more likely to seek help from military charities, and this is consistent with previous research [7].

The most common type of need presented by veterans was social wellbeing, followed by physical health. Only a small percentage presented with a mental health need, while other studies report much higher prevalence of these conditions [5, 6].

Several characteristics that may indicate that veterans are a vulnerable population were identified. In of detail, the repetition of access, the receipt of other benefits, and the living conditions of some veterans. In particular, the fact that 39.9% of veterans accessed charity services more than once suggests that their needs are not fully met in the first access. Furthermore, 67% of them received other forms of benefits, suggesting that the need for support is often not limited to a simple need, but probably part of a more complex problem [23]. This suggests that the veteran population may have a high level of complexity [31, 32], and this is consistent with previous studies [5], where veterans were found to be resistant to ask for help until the problems become multiple and unmanageable.

In addition, a significant percentage of the veterans who accessed charity services were living in prison or were homeless, indicating a severe form of difficulty. The fact that 25% of people who accessed had dependants (children or spouses) may indicate that the number of those in need might be even higher than what reported from sector charities.

The stratification ‘*a priori*’ based on usage pattern showed statistically significant differences among subgroups. Specifically, the subgroup with a single access and a single need had a higher frequency of being married and a low percentage of other benefits’ claim. This is consistent with previous literature [33] and could be attributable to the fact that those with a stronger family support net tend to go less frequently in crisis. It is therefore possible that this subgroup is the one with the lowest complexity, having stronger family support and less need for charitable services.

On the other hand, the subgroup with multiple accesses presenting with all different needs was the one with the lowest age and lowest prevalence of officers. In addition, this group had the highest rates of people living in prison or being homeless and the highest percentage of people asking for other kind of benefits. These characteristics corroborate the assumption that this subgroup is the one with the highest complexity, probably suffering from issues that cannot be solved by a charity service but probably need a more holistic evaluation. In fact, it is possible that younger veterans with lower ranks have a lower education level [34], which is a recognized social determinant of health [9].

However, the differences among subgroups are modest in absolute terms, indicating that the veteran population who accessed charity services is relatively homogenous. This may suggest that the factors that influence the usage of military charity services could be related to other variables that were not included in our study. Future research should explore other possible determinants and their associations with the usage of charity services by veterans.

### Strengths and limitations

One of the main strengths of this study is that it provides the first comprehensive registry of veteran charity usage data in the UK. This is the first study that combines data from multiple charities that offer different types of support to veterans, applying a set of definitions and harmonizing heterogeneous datasets. This approach allowed us to gain a deeper understanding of the characteristics of the veterans who access charity services, and to identify patterns that may not be evident from a single dataset.

Another strength is that for the first time in the UK it includes a high number of veterans who accessed military charity services. This number can be considered reliable, as double counting people who were helped by multiple charities, (a process called ‘almonisation’, where multiple charities financially contribute to the resolution of a single problem) was eliminated. Prior to this study, each charity involved in an ‘almonised’ case would record that case as an individual helped, therefore, a person was counted multiple times for the same problem. In the military charity sector, this created a significant inflation of usage rates, since the datasets were not linked with proper caution. This process was made possible by the use of SHA as pilot tested by Tomietto *et al.*, ensuring data anonymity and improving the unique identification of each veteran across different data sources [20]. The aggregated dataset has also the potential to be linked to other large datasets (e.g. from NHS registries) to provide a wider understanding of the health and social care needs of this population.

One of the main limitations is the coverage of the charity sector in the UK. Our study is not yet fully inclusive of the whole military charity sector. Therefore, our results may not reflect the full spectrum of services that are used by veterans in the UK and may be biased towards the types of services that are offered by the charities in our study. In the future, the MONARCH project will expand the coverage of the charity sector and include data from more charities that provide different types of support to veterans.

Another limitation of this study is the data incompleteness. Not all the charities in our study provided data on all the variables that were analysed, and this may constitute a source of bias. To address this issue, feedback was provided to charities sharing data, and a working group on data strategy was set up. It is, therefore, expected that in following years, the quality of data will improve constantly.

A further limitation of this study is the absence of a control group, as we did not compare the veterans who accessed charity services with those who did not. This limits our ability to identify the factors that determine the usage of charity services by veterans. Future research should focus on recruiting an appropriate a control group to conduct a comparative analysis.

Finally, the machine learning approach should be both considered as a strength and a limitation. The ‘unsupervised’ automated approach returned the same results as the ‘*a priori*’ approach, strengthening the validity of the hypothesized pattern. However, the K-means algorithm needs a careful variable selection to provide useful insights, and, if this condition is met, the results are similar to those obtained using an approach ‘*a priori*’.

## Conclusion

This study provides a novel and comprehensive analysis of the usage of charity services by veterans in the UK. This work established, for the first time in the sector, a registry of veterans and a methodology for data aggregation and harmonization based on a shared definition of health and social needs. This study provides useful insights to decision-makers and may be used to guide future planning and health and social care provision. Future developments will expand the registry by including a wider consortium of charities and involving the NHS. This will strengthen the study of the determinants of service usage and to uncover the causal pathways leading veterans to a crisis.

Conflict of interest: None declared.

## Data Availability

The data supporting this study's findings are available on request from the corresponding author. The data are not publicly available due to privacy or ethical restrictions.
